# Dancing with atrial fibrillation – How arrhythmia affects everyday life of family members: A qualitative study

**DOI:** 10.1371/journal.pone.0254130

**Published:** 2021-07-06

**Authors:** Stine Rosenstrøm, Signe Stelling Risom, Camilla Ejlertsen, Jens Dahlgaard Hove, Anne Brødsgaard

**Affiliations:** 1 Department of Cardiology, Copenhagen University Hospital, Amager Hvidovre, Hvidovre, Capital Region of Denmark; 2 Department of Public Health, Nursing and Health Care, University of Aarhus, Aarhus, Denmark; 3 Department of Cardiology, Herlev and Gentofte University Hospital, Hellerup, Denmark; 4 Institute of Nursing and Nutrition, University College, Copenhagen, Denmark; 5 Copenhagen University, Faculty of Health and Medical Sciences, Copenhagen, Denmark; 6 Department of Gynecology and Obstetrics, Copenhagen University Hospital, Amager Hvidovre, Hvidovre, Capital Region of Denmark; Maastricht University Medical Center, NETHERLANDS

## Abstract

**Background:**

Atrial fibrillation (AF) is the most common cardiac arrhythmia. Patients with AF often experience debilitating symptoms, stress and reduced health-related quality of life. Previous qualitative research on AF has primarily focused on the patient. AF, however, can also be burdensome for the patient’s family.

**Aim:**

The aim of this study was to explore how family members experience life when a close member in the family has AF.

**Method:**

Transcribed focus group interviews were analysed using content analysis approach inspired by Graneheim and Lundman.

**Results:**

Two focus group interviews were conducted with 11 family members. The overall theme was *Dancing with AF*. The theme emerged from three categories: *1***)** *Handling AF as a living condition*, *2) Influencing the roles of family members*, *3*) *Fear of AF attack*. AF had a very significant impact on the patients’ family members, forcing them to reconstruct their daily lives.

**Conclusions:**

AF has multiple consequences for family members and can give rise to conflicts concerning family roles. Family members have a lack of knowledge of AF and fear of how AF can cause changes in the family members’ everyday lives. This study demonstrates that there is a need for further research of ways to support the family members of patients with AF.

## Introduction/background

AF is the most common cardiac arrhythmia. An estimated 33.5 million people live with AF worldwide, with higher incidence and prevalence in the developed countries [1]. Patients with AF often experience debilitating symptoms, including palpitations, chest pain and dizziness [2]. These symptoms can cause psychological stress and reduced health-related quality of life [3], thus burdening both the patients and their families [4, 5]. Clinical guidelines recommend family involvement to encourage self-management, adherence to therapy and empowering patients to facilitate participation in shared decision making [2, 6]. However, little attention has been devoted to interventions involving family members in patient education and managing everyday life with AF. Managing life with a chronic illness can be difficult, not only for the patient, but also for family members [7, 8]. Finding support for family members involved in the caring of patients with heart disease needs to be included in the health care plan [9, 10]. Furthermore, many patients and families affected by chronic illness seek a return to normal and wish to regain balance in their daily lives [11].

Several qualitative studies have focused on the patient with AF [12–14]. Quantitative studies have also shown how patients with AF struggle with impaired health-related quality of life, stress and anxiety due to AF symptoms [15]. A study found that AF patients and their spouses influence each other and that higher levels of anxiety and depression were associated with lower levels of perceived health for both parties [5]. Living with and adjusting to life with a chronic illness is a family affair and [16]) mandates that research be devoted to exploring the family perspective [17, 18]. Modern family structures can be diverse: in this study we let the patients themselves point out their “close one” and accordingly define family as *“family is who they say they are”* [19]. The aim of this study was to explore how family members experience life with a close one in the family who has AF.

## Method

### Design

In this inductive explorative qualitative study, data were gathered through focus group interviews [20], a method which allows the facilitation of the perspectives of the family members confronted with AF. An inductive approach can be useful in cases where no previous studies have confronted the research question [21]. The inductive process moves from specific observations to broader generalizations. The COREQ (COnsolidated criteria for REporting Qualitative research) checklist was followed through developing, performing and reporting the study [22].

### Data collection

Family members, were by convenience sampling, [23] recruited through patient contacts in a Copenhagen University Hospital Amager Hvidovre, located in the Capital Region of Denmark. When a patient with AF had a face to face nurse consultation at the outpatient clinic in the Department of Cardiology he/she was asked if we were allowed to contact a close family member chosen by the patient. Thus, the inclusion criteria were: 1) being a close family member to a patient with AF regardless of type of AF, burden of AF and treatment of AF, and without heart failure, diagnosed according to the European Society of Cardiology (ESC) guidelines [2] and 2) the family member had to be ≥ 18 years old. Exclusion criteria were substantial language barrier and cognitive impairment.

A semi-structured interview-guide (S1 File) was developed based on literature living with AF and how it affects family members. The opening questions were”What´s it like living with a person who has AF?” followed by “In what way does AF affect your life as a family member?”. The two focus group interviews were conducted in October 2019 at the University Hospital, in a private meeting room. Both focus group interviews were facilitated by the first author (primary interviewer) and the last author (facilitator) who had no prior knowledge about the family members. The role of the primary interviewer was to follow the interview guide questions and try to make the family members feel free and open to discuss their feelings and experiences. The role of the facilitator was to support the primary interviewer in keeping track of the interview questions, time frame and to observe the reactions, verbal communication and body language between the family members. This was firstly to ensure that all family members were able to speak up about their unique experiences and secondly to make sure that not one person in the group was to dominant, making it possible to get nuanced experiences in order to illuminate the aim of the study. The first author was a PhD student with 20 years of experience as a clinical nurse in cardiology and last author was an associate professor with more than 10 years of experience in qualitative research methods. The atmosphere between the family members was relaxed and they were all positive to have been invited for the focus group interview. The family members responded with openness and willingness to discuss the focus of the study and they were engaged and willing to talk to each other. The dynamics in both interviews were sometimes intense as some of the family members had many feelings to express. Both focus group interviews were digitally recorded and transcribed verbatim by the first author. All data were stored and analyzed in Nvivo12 PRO [24]. In total, sixteen family members were asked to participate and accepted. However, four family members canceled due to illness and one because she reconsidered and discovered that she felt uncomfortable talking about their situation. As recommended in the literature, the goal was to gather an optimally-sized focus group of six to eight participants [23].

### Data analysis

A conventional content analysis method inspired of Graneheim and Lundman was used to analyze the data [21]. The analysis involved a reflexive process described in six steps. The analysis focused on the latent content which is the underlying meaning of the content which requires some levels of abstraction. First, a naive reading of the interviews was performed several times to obtain a sense of all the material. Secondly, the text was sorted in to one text about the family member experiences. Third, the text was divided into meaning units and condensed, which is a constellation of words or statements related to the aim of the study. Fourth, the condensed meaning units were labeled with codes. Codes can be assigned to objects, events or other phenomena and the understanding cannot be separated from the context. A discussion of codes related to the overall context was done in collaboration by the first and third authors. Fifth, various codes were compared, based on differences and similarities and sorted into sub-categories and categories. A process of reflection and discussion resulted in the final sorting of the codes by the entire research team. Sixth, the content was formulated into an overall theme [21].

### Rigor

To meet the criterion trustworthiness in qualitative research the following concepts were followed: credibility, transferability, dependability and confirmability [21]. To ensure credibility, the chosen research question, data analysis, and context for the focus group interviews were performed in collaboration with all authors who have various competences in research methods and cardiology. To ensure transferability representative citations from the transcribed text were included to support the findings. Furthermore, the findings were thoroughly discussed within the research group until agreement. Dependability and confirmability were obtained through the interviewers’ ability to facilitate positive dynamics into both focus group interviews and to motivate the family members to talk about their experiences. Furthermore, the first author was interviewed about her preunderstanding before interviewing the family members. This was done in order to prevent unacknowledged preconceptions related to the focus of the study and thereby increase rigor of the project [25].

### Ethical considerations

The study conforms with the principles outlined in the Declaration of Helsinki [26]. All family members received written and oral information about the study and written informed consent was obtained. Data were handled, processed and analyzed, maintaining confidentiality, and then anonymized. The study was approved by the Danish Data protection Agency (VD-2019-42) and the Local Ethical Committee, Capital Region of Denmark (id: 19007769).

## Results

The focus group interviews resulted in a total of 11 family members in the following relationships with the patients: male partner, female partner, mother, or daughter (Table 1). The focus groups consisted of, respectively, five and six family members. Their close ones had the AF diagnosis for a mean value of 1.5 years (range 2 months– 6 years). The close ones with AF was described by the family members to have had several periods since diagnosed with AF with many symptoms causing lack of energy that interrupted activities in the family. All most all the close ones with AF were described as having tried one or several medicaments for rhythm control, and some had also tried electrical cardioversions. At the time of the interviews none of the close ones with AF were referred to ablation according to the family members knowledge. Data on burden of AF symptoms, duration time of AF and type of AF were based on information from the family members and measured by the European Heart Rhythm Association symptom classification for atrial fibrillation (EHRA-score) [2], which is a validated instrument for measuring and monitoring symptoms in the management of AF care (Table 2).

**Table 1:** Characteristics of family members.

Family relation to patient with AF	n
Male partner (n)	4
Female partner (n)	5
Daughter (n)	1
Mother (n)	1
Living together with a patient with AF	9
Age (Range/mean)	39-75/63.5 years

**Table 2:** Characteristics of AF burden in the close one with AF (the patients).

Patients with AF (the close ones)	
Age Range/Mean	21-80/67.5
Male (n)	7
Female (n)	4
Time since AF diagnose (Range/Mean)	2 months- 6 years/ 1.5
*EHRA Score (Range/Median)	1–4 (2b)
Type of AF (n)	
Persistent (n)	6
Paroxysmal (n)	4
Permanent (n)	1

* EHRA-score: European Heart Rhythm Class symptoms: 1 (no symptoms) AF does not cause any symptoms. 2a (Mild symptoms) normal daily living not affected by symptoms related to AF. 2b* (Moderate symptoms) normal daily activity not affected by symptoms related by symptoms (but patient troubled by symptoms). 3 (Severe symptoms) normal daily activity affected by symptoms related to AF. 4. (Disabling symptoms) normal daily activity discontinued [2].

The overall theme that emerged from the content analysis was: *Dancing with AF* describing the family members´ experiences and reflections. It emerged from three categories and nine sub-categories (Table 3). The categories: *handling AF as a living condition; influencing the roles of family members; fear of AF attack* are elaborated in the following and supported by citations. X indicates a female and Y a male family member, and the numbers refer to the anonymized family member.

**Table 3:** Themes, categories, and sub-categories.

Theme	Categories	Sub-categories
**Dancing with AF**	Handling AF as a living condition	Life with AF has consequences for social activities
		Concerns for worsening AF
		Family rituals and routines create security
	Influencing the roles of family members	Validate knowledge and feelings in interaction with their close one
		The interaction is changing
		Family members like watchdog
	Fear of AF attack	Lack of knowledge about AF
		Underlying insecurity
		Limited options for actions

### Dancing with AF

The overall theme showed that family members observe, support and adapt to their close one with AF. Family members have constant conscious and unconscious exchanges of information in interaction with their close one who has AF. It is as if the family member and the close one move together in a dance. Occasionally, the dance requires a family member to take the lead, but at other times AF determines the rhythm of the entire family, depending on whether the symptoms of AF are being experienced by the patient or activities are planned around the fear of symptoms arising.

### Handling AF as a living condition

Family members experienced disruption in family life when a close one had AF. Life with AF could play havoc with planned social activities in the family. Family members said that AF often caused fatigue and depletion of energy in the patient with AF. Several family members had mentioned that it could be very demanding for them when AF took all the energy out of their close one with AF, and therefore, the family members often had to take more responsibility for tasks at home. As examples, family members recalled sometimes having to cancel social activities with other family members, friends and traveling plans. Similarly, gardening and home improvement projects could get lower priority depending on the level of energy and the physical wellbeing of the patient, which could cause irritation and frustration in the family. Some family members felt that AF could make the patient feel very exhausted after social activities, resulting in guests being invited to their home less frequently than before. AF forced adjustments for the whole family; forcing both family member and the close one with AF to think more about how to prioritize their energy.

*“We have not had a large dinner party…*‥ *If we have guests*, *then the next day he´s exhausted*. *Then he has to remove one or two days from the calendar because he’s burnt out*. *So atrial fibrillation gives us some limitations and quite a few restrictions*. *We have been thinking a lot about this really”*. X-1

Another family member in the focus group said:

“*It is also our problem*, *I have told him that we need to move to an apartment with less work to do*, *but the garden means so much to him and he is not ready to make the decision yet*, *so we will see*…*” X-2*

Family members to a close one with AF often had several concerns for worsening AF. The illness reminded the family members that life does not last forever and that there could be a risk that AF symptoms could worsen. The fact that many of the family members were getting older meant that there were people in their entourage who had died from heart disease. Family members who knew someone who had died from heart disease experienced anxiety that AF could cause a heart attack. Several family members experienced feeling touchy and having a need for being in control over what might happen to their close one with AF. Some experienced anxiety or got nervous when the close one with AF was delayed in their return to home and they could not get hold of him or her. Several alarming thoughts could arise about what might have happened. For some, the mobile phone became a lifeline, knowing that they could always get in touch with each other.

*“It is probably me who has become touchier about it*. *I have given him a mobile phone and told him to call if he is delayed”*. X-2

Family members created new routines such as telephone chains which they could activate in an acute situation. This helped them to know exactly whom to contact in case of hospitalization. Furthermore, some family members had small rituals, such as serving a hot cup of tea and fetching a warm blanket when their close one had an AF attack.

*“Faith can move mountains*. *I have been making milk for him lately before going to bed and he thinks it helps a little*. *And it may well be that it’s just faith*. *But he thinks it helps him to calm down when he wakes up due to a pounding heart”*. X-3

Routines and rituals resulted in the family member and the close one feeling more secure and in control with the unpredictable symptoms from AF.

### Influencing the roles of family members

The roles in the family were affected when the patient was diagnosed with AF. This was very clear to the family members in situations with new treatment or a hospitalization.

Family members validated their knowledge and feelings in interaction with their close one. This meant that family members felt that they had an essential role in validating and asking questions about the information that came from the health professionals and especially if the patient with AF felt nervous or anxious about AF. This could result in family members often having to repeat information and treatment plans given by the hospital to the close one with AF. Therefore, it was important to many family members to join their close one when going to the follow-up consultations at the hospital. When the close one with AF did not involve the family member in the information from the hospital about the treatment or other important information regarding AF, it left the family member worried with a feeling of being left outside. Furthermore, the family members also experienced a need for being involved in order to strengthen their own ability to cope with the many thoughts, questions and worries that could arise when a loved one (partner, parent or child) was diagnosed with AF.

*“Initially*, *my husband did not want to take me to the consultations (in the hospital)*, *but then I insisted that I should come with him*. *I told him that I needed to talk to them (nurses and doctors) about how I really felt and how it also affected me”*. X-4

Some family member described how they often had to be the one who repeated what the health professionals at the hospital had said, because the close one with AF did not understand why he or she had recurrent palpitations due to AF. It could be frustrating for the family members not being able to prevent limiting symptoms and side effects from AF and its treatment.

*“He is so disappointed when AF comes again*, *and I try to tell him that the nurse said it is something that can keep coming and probably also increase in intensity over time*.*”* X-5

The interaction in the family changed due to AF. Family members told how interactions and roles in the family were affected by AF. Thus, they had ambivalent feelings about the level of their involvement in the treatment or they sometimes insisted on going to the hospital to a medical consultation about AF. In addition, several family members felt sorry for the close one with AF when they experienced symptoms. Some family members had to be aware of their own needs and continue to do things such as participating in social activities and exercise for their own well-being and health. This could be necessary when the close one with AF was no longer capable of participating in social activities that required more strenuous physical effort.

*“He can tell that I’m sorry and upset*. *I can also feel and see when he is not feeling well*, *which is most of the time*. *It’s a pity for him and I am sorry for him*. *I can do nothing but make him stay home and then I drive off to some event*. *Because he can’t make it”*. X-6

Several family members experienced that they felt a need to monitor the close one with AF, especially in the beginning. The family members felt they were like watchdogs. This was mirrored by how family members began to observe the other, both verbally and non-verbally, by noticing movements and the tone of voice. Some had a direct approach when asking how the other was doing, while others described themselves as watchdogs, observing signs of the other person’s wellbeing. Some of the family members had a great need to be in control over the situation and felt that they had to be protective if they had a feeling that there was something wrong.

*“I know when there is something wrong and I am like a machine because I confront him*. *But I know that he is also someone who doesn’t wish to talk about it much*, *and I don’t leave him alone until he speaks up about how he is”*. X-7

Family members felt in different degrees and in various ways how AF had an impact on their roles in the family. When interviewing family members, the effect of AF became clear to some of them. This is the first time these family members had reflected on these issues, with anyone other than themselves.

### Fear of AF attack

Most of the family members, regardless of their close one having heavy symptom burden from AF or only few symptoms, felt they had a lack of knowledge about AF; this being particularly difficult in the first months after their close one had been diagnosed with AF. A few family members had heard something about AF before their close one had AF. However, the majority did not have any knowledge at all; this made them feel very insecure if anything unpleasant should happen with their close one. Several family members described how they had a strong desire to know what could keep AF under control or to keep symptoms to an acceptable level for their close one.

Therefore, most family members had a great desire for more information, guidance and useful advice on how to prevent limiting symptoms from AF. Although several of the family members managed, over time, to familiarize themselves with AF and its nature, many family members felt an underlying insecurity.

“*After all*, *there is no doubt that you*, *as a family member*, *are affected by AF*. *When your wife*, *whom you have known for most of your life*, *has severe symptoms*, *and it becomes quite clear that she is not well when she gets the attack* … *you can´t avoid being worried and thinking about the consequences of what will happen if help doesn´t arrive”*. Y-8

Another family member responded:

*“It is basically because we are afraid that they can die*, *we are afraid to lose our loved ones”*. *X-10*

Family members often felt that they had limited options to help their close one due to their little knowledge of AF. Family members required more information about the arrhythmia and about which daily life things might trigger AF attacks if it was a close one with many periods with symptoms from AF.

*“Well*, *there’s so much*. *He thinks a lot about what it is that triggers it and what he can do to prevent it*. *I know what the answer is*: *No*, *you can’t do anything*. *But it would be nice to have some advice*!*”*. *X-9*.

Family members expressed how they had limited options for action toward AF. This was regardless of their close one had felt symptoms from AF as a heavy burden. Most of the family members expressed a need to be more involved with information and patient education regarding symptoms of AF, medication, exercise and nutritional advice. This could make them feel less insecure and have less fear of AF. All the family members felt a great need to be supportive to their close one.

## Discussion

The overall theme *Dancing with AF* and the findings demonstrated that AF often had a tremendous impact on family members’ everyday lives. Being a family member to a close one with AF resulted in several adjustments in daily life. The findings highlighted some of the challenges that family members of patients with AF needed to manage. Also, families confronted with chronic illness can benefit from support by healthcare professionals to become aware of existing patterns in the family and to develop new coping strategies [7]. Thus, this area of nursing could potentially, if focused upon, increase the families’ coping skills in the setting of a chronic arrhythmia disorder like AF.

The finding *Handling AF as a living condition* revealed how family members had to cope with the fact that AF was now a part of their lives. A qualitative study with patients suffering from very symptomatic cardiac arrhythmias, including AF, also found that the burden of symptoms had a great impact on family members [27]. Symptoms could place a substantial pressure on the relationship between the patient and family member, and some families even stopped taking care of their grandchildren. They could feel guilty about having to ask for help from friends and family ultimately leading to isolation for the patient and the family [27]. Similarly, we found the same pattern in the family members’ thoughts about living with AF in the family although to a lesser degree. This difference in the findings could be caused by the high symptomatic burden in the patients examined in the study by Withers et al. as these patients were scheduled for invasive treatment [24], whereas our related patient group was followed in a standard care out-patient clinic focusing on medical anticoagulation treatment and rate control.

Also, consistent with our findings, the study by Coleman et al. demonstrated that it was a major burden to family members of patients with AF when the arrhythmia caused disruptions in their plans and schedules [28]. They found that medication issues appeared to be a notable burden to the family members. On the contrary, the issue of insecurity about medication was not brought up by the family members in our study. Nevertheless, it is important to ensure that family members have some level of knowledge to support the patient in taking the medication correctly [29].

The finding *Influencing the roles of the family members* revealed how AF affected the roles of each member of the family. This finding could be useful in planning future AF education programs by placing a greater focus on the family members’ experience and their need for illness-specific education. This has already been proposed by Kokorelias et al. who have identified core aspects of family focused care such as illness specific education. Furthermore, the need to focus on both the patient and the family members was mirrored in a study of patients’ and professionals’ experiences of pilot-testing a Learning Café group education program for patients with AF and family members [30]. The study demonstrated that patients´ overall sense of security increased after the session. However, the study did not focus on the family members as they were not a part of the interview study evaluation. In future education and support for patients with AF and their family members, it is vital to have some evidence of effective ways to involve and integrate the family members [31].

The finding *fear of AF attack* revealed that AF had impact on the everyday lives of the family members. Research has demonstrated that AF not only affects the AF patient, but it also impairs the quality of life of the spouse to a similar degree [32]. These results are in line with our findings which reflect that supporting family members could be beneficial in an integrated care program for patients with AF [2, 6]. Furthermore, family health conversations have shown positive results supporting patients with a chronic illness and their family members by offering them the opportunity to constitute self-identity and identity within the family, increasing the families’ understanding of their own resources and multiple ways of acting and seeing new possibilities [17, 33]. Potentially, family focused interventions could improve the well-being of AF patients’ family members. In line with this, the ESC guidelines recommend involving family members in care and decision-making [2]. However, no concrete interventions or ways to involve family members are described in the guidelines.

Overall, the theme and category findings in our study can be mirrored to the findings found of Ekblad et al. who in 2014 almost had similar results in their qualitative study based upon individual interviews with family members to AF patients [34]. Therefore, we argue it is now time to actively focus on the family perspective and integrated care in AF nursing, and to develop and test the needed intervention tools to facilitate this process.

### Limitations

The size of the focus group in one of the two interviews was small, having only five family members, which could potentially limit data saturation [23]. However, the use of a qualitative methodology with focus group interviews has provided us with rich data on how life with AF is experienced by family members. Most of the family members recruited for the focus group interviews were partners and therefore the study mostly presents the perspective from the caregivers in the close family relation and not the extended family situation. We found it more challenging to recruit friends and adult children for an interview at the hospital because of the greater physical or social distance arising when they were not living together. The risk of information bias potentially exists. Firstly, the included family members were the ones most willing to participate in a focus group interview. Secondly, the family members who did not attend the focus group interviews may have had other experiences of being a family member of a patient with AF. However, these focus group interviews were conducted by a primary interviewer with specialist knowledge in cardiology, which enabled her to pursue the various experiences of these unique family members. The facilitator had extended methodological knowledge and had facilitated many focus group interviews, which made it possible to achieve maximal utilization of the group dynamics and pursue statements from the family members.

The interviewers complemented each other and achieved rich and nuanced data. The lively group dynamic made the family members very interested in sharing their experiences. To ensure reflexivity and preventing the first authors´ preunderstanding from influencing the findings, the coding process and the coding per se were discussed several times between the research groups until an agreement was reached. The family members described their close ones having periods with limiting symptoms. This qualitative focus group study focused on the experiences of the family members. Though, it would be relevant to discuss the role of appropriate referral for AF treatment helping both patient and family member. Thus, it would require a different design, with a larger sample of patients and families to be able to look at any association between how families cope with AF according to treatment and burden of symptoms. Furthermore, we were not able to determine whether the impact on the family members daily lives was related to the close ones burden of AF, thought the arising themes point in that direction.

The essence of the findings in this study could be transferable to other family members of patients living with a heart disease and not only limited to family members close to a patient with AF.

## Conclusion and perspectives

In conclusion, having a close person in the family with AF has multiple consequences for the family members. The theme *“dancing with AF”* is a metaphor for how family members follow the patient with AF and how family members need to learn a new dance which often requires new knowledge and renewed family roles. Family members can potentially benefit from family focused interventions. Additional knowledge is needed to examine how healthcare professionals can guide and support family members in order to adjust and manage life with a close one having AF.

Future research should address interventions targeting family members.

## Supporting information

S1 File Interview guide used for focus group interviews. (DOCX)

S1 Checklist (PDF)
