# Supplementary material for: Dancing with atrial fibrillation – How arrhythmia affects everyday life of family members: A qualitative study
Source: PLoS One. 2021 Jul 6;16(7):e0254130. doi: 10.1371/journal.pone.0254130 (PMC8259977; doi:10.1371/journal.pone.0254130)
Supplement: S1 File — (DOCX) [file pone.0254130.s001.docx]

**Fokusgruppe interviews**

***Hvordan oplever familiemedlemmer livet med en pårørende der har atrieflimren (AF)?***

| **Kernespørgsmål** | **Uddybende spørgsmål** |
| --- | --- |
| Præsentation af deltagere:   1. **Hvordan opleves det at være pårørende til en der lever med AF?** 2. **På hvilken måde indvirker sygdommen jeres liv som pårørende?** 3. **Er der nogle særlige følelser der er i spil hos jer-**   **- og er det de samme som hos jeres familiemedlem med AF?**     1. **Har sygdommen betydet at I skulle ændre jeres hverdag?** 2. **Hvis der er ét spørgsmål, I gerne ville have svar på, hvad vil det så være?** 3. **Hvilken støtte har I som pårørende oplevet at få fra familie? Den pårørende med AF? Venner?** 4. **Hvordan vil I gerne have jeres familiemedlem inddrager jer i dennes sygdom?** 5. **Hvordan vil I gerne inddrages af de sundhedsprofessionelle?** 6. **Hvad tænker I om fremtiden mht. hvordan AF påvirker jeres familie?** 7. **Interventionen-Hvad tænker I om den?** | Hvordan påvirker AF jeres liv?   - Kom med konkrete eksempler?   Har I nogle bekymringer?  Beskriv hvilke og hvordan det konkret kommer til udtryk?  Hvilke tanker gør I jer om sygdommen AF?  Hvordan kommer jeres følelser til udtryk?  Er det helt naturligt for jer at tale med hinanden om hvordan I har det?  Er der ting som er svære at tale om eller spørge om som pårørende?  Har I selv opsøgt viden og evt. hvor?  Hvilke tilbud kunne I ønske jer om information?   - Sætter I begrænsninger for jeres familiemedlem med AF?   Fortæl evt. med jeres ideer til hvordan pårørende kunne inddrages? I samtaler og undervisning?  Hvordan ser det ud om en måned eller om fem år? Hvad ønsker I jer for jeres familie? |
| **Tak fordi I kom og TUSIND tak fordi I delte jeres oplevelser og historier med os. Det har stor betydning for vores kommende arbejde og forhåbentlig for de kommende patienter og deres familier.** | **Hvis I får nogle spørgsmål eller kommer i tanke om noget er I velkommen til at kontakte Stine på mail eller tlf.** |

**Focus group interviews:**

***How do family members experience life with a relative who has atrial fibrillation (AF)?***

| **Core issues** | **Elaborating questions** |
| --- | --- |
| **Presentation of participants:**  **1. How does it feel to be a relative of someone living with AF?**  **2. In what way does the disease affect your life as a relative?**  **3. Do you have any special emotions at stake?**  **-and are they the same as with your family member with AF?**  **4. In what way has the illness caused any changes for your everyday life?**  **5. If there is one question you would like answered, what would it be?**  **6. What support have you as a relative experienced receiving from family? The relative with AF? friends?**  **7. How would you like your family member to involve you in his or her illness?**  **8. How would you like to be involved by the health professionals?**  **9. What do you think about the future in terms of how AF affects your family?**  **10. The intervention- What do you think about it?** | How does the AF affect your life?   - Come up with concrete examples?   Do you have any concerns?  Describe which and how it is concretely expressed?  What thoughts do you have about the disease AF?  How are your feelings expressed?  Is it completely natural for you to talk to each other about how you are feeling?  Are there things that are difficult to talk about or ask about as relatives?  Have you sought out knowledge yourself and possibly where?  What kind of information would you like to be offered?  - Do you set limits for your family member with AF?  Tell us your ideas on how relatives could be involved? In conversations and possibly in education?  What does it look like in a month or in five years?  What do you want for your family? |
| **Thank you for coming and THANK YOU so much for sharing your experiences and stories with us. It is of great importance for our future work and hopefully for the future patients and their families.** | **If you have any questions or come up with something, you are welcome to contact Stine by email or tel.** |
